# Sarsaparilla (*Smilax Glabra* Rhizome) Extract Inhibits Migration and Invasion of Cancer Cells by Suppressing TGF-β1 Pathway

**DOI:** 10.1371/journal.pone.0118287

**Published:** 2015-03-05

**Authors:** Tiantian She, Chuanke Zhao, Junnan Feng, Lixin Wang, Like Qu, Ke Fang, Shaoqing Cai, Chengchao Shou

**Affiliations:** 1 Key Laboratory of Carcinogenesis and Translational Research (Ministry of Education), Peking University Cancer Hospital & Institute, Beijing, China; 2 State Key Laboratory of Natural and Biomimetic Drugs, School of Pharmaceutical Sciences, Peking University, Beijing, China; Seoul National University, KOREA, REPUBLIC OF

## Abstract

Sarsaparilla, also known as *Smilax Glabra* Rhizome (SGR), was shown to modulate immunity, protect against liver injury, lower blood glucose and suppress cancer. However, its effects on cancer cell adhesion, migration and invasion were unclear. In the present study, we found that the supernatant of water-soluble extract from SGR (SW) could promote adhesion, inhibit migration and invasion of HepG2, MDA-MB-231 and T24 cells *in vitro*, as well as suppress metastasis of MDA-MB-231 cells *in vivo*. Results of F-actin and vinculin dual staining showed the enhanced focal adhesion in SW-treated cells. Microarray analysis indicated a repression of TGF-β1 signaling by SW treatment, which was verified by real-time RT-PCR of TGF-β1-related genes and immunoblotting of TGFBR1 protein. SW was also shown to antagonize TGF-β1-promoted cell migration. Collectively, our study revealed a new antitumor function of Sarsaparilla in counteracting invasiveness of a subset of cancer cells by inhibiting TGF-β1 signaling.

## Introduction

Sarsaparilla, also known as *Smilax Glabra* Rhizome (SGR), is a natural dietary supplement widely used in food-making and health care, based on its capability in detoxicating, clearing heat and relieving dampness [1,2]. Some SGR-containing beverages, foods and dietary supplements are purchasable in Southeast Asia and Northern America. Patients with dermatitis, syphilis or gouty arthritis in Southeast Asia have benefited from the treatment of SGR-containing herbal mixtures for a long history [3,4]. Currently there are also growing scientific evidences reporting its therapeutical potential for the treatment of rheumatoid arthritis [5], inflammation [6], liver injury [1], hyperinsulinemia [7] and cancer [8].

The functions of SGR are mainly divided into four aspects, namely immunomodulatory, hepato-protective, tumoricidal and others. On the immunomodulatory aspect, the aqueous extract from SGR exerts a marked inhibition on picryl chloride (PCl)- or sheep red blood cells (SRBC)-induced delayed-type hypersensitivity (DTH) [6,9]. SGR primarily acts on cellular immune response (CIR), the effector phase of DTH rather than humoral immune response (HIR), thus conferring SGR a superior advantage to other immunosuppressors in treating CIR-mediated inflammatory diseases like hepatitis and rheumatoid arthritis [6,9]. Astilbin, one of bioactive compounds isolated from SGR, can alter the *in vivo* cytokine profiles of lymphocytes and suppress the migration of activated T cells, thus relieving contact hypersensitivity and DTH [10,11]. On the hepato-protective aspect, Astilbin facilitates the apoptosis of the liver-infiltrating T lymphocytes and inhibits the cell-matrix adhesion of splenocytes to minimize liver damage [12,13]. Furthermore, it could improve the liver function by reversing transaminase elevation, lowering TNF-α production and reducing the hepatotoxicity of nonparenchymal cells [12,14]. Taxifolin, another compound isolated from SGR, was found to change lipid metabolism to relieve liver burden [15,16]. The function of SGR also extends to other biological functions, including repressing helicobacter pylori activity [17], lowering the blood glucose [2] and reducing activity of HIV-1 integrase [18]. All these findings point to the multifunctional potential of SGR.

On the anticancer aspect, oral intake of a herbal formula containing SGR was found to extend pain-relieving sustained time, improve patients' quality of life and prolong long-term survival of patients with hepatic carcinoma [19]. Another SGR-containing injection was discovered to decrease tumor growth at relative high doses in mice models [20]. Extracts from SGR were found to promote apoptosis in human colorectal cancer HT-29, human hepatic cancer HepG2 and HepG3 cells [8,21]. There are also several hints indicating the possible roles of SGR in controlling cell adhesion and migration. Astilbin can suppress the adhesion of splenocytes to extracellular matrix in liver-injured mice models [14], and block intercellular adhesion between human Jurkat T cells and ECV-304 cells [13]. In addition, 5-O-caffeoylshikimic acid, taxifolin and astilbin from SGR inhibited the migration and adhesion of macrophages [22]. Nonetheless, the direct role of SGR extract on cancer cell invasiveness is unclear and the mechanistic basis is lacking. In the present study, we evaluated the effects of the supernatant of water-soluble extract of SGR (SW) on the adhesion, migration and invasion of three cancer cell lines, and explored the possible mechanism.

## Materials and Methods

### Ethics Statement

Animal study was approved by the Biomedical Ethical Committee of Peking University Cancer Hospital & Institute and performed along established institutional animal welfare guidelines concordant with the US guidelines (NIH Publication #85–23, revised in 1985).

### Materials

Matrigel was purchased from BD Biosciences (San Jose, CA). Antibodies to vinculin and TGFBRI were purchased from Sigma-Aldrich (St. Louis, MO) and Bioworld (Beijing, China) respectively. TGF-β1 was from Sigma-Aldrich.

### Preparation of SGR extract

SGR were obtained from Ben Cao Fang Yuan Pharmaceutical Co. (Beijing, China). The procedures for preparation of the supernatant of water-soluble extract from SGR (SW) were described previously [23]. The yield percentage for SW was 7.27% (g/g). The solvent for preparation of SW (stock solution) was 3% DMSO in PBS, and the DMSO in working solution of SW was lower than 0.15% (v/v).

### Cell culture

HepG2, MDA-MB-231 and T24 cells were obtained from ATCC (Rockville, MD) and cultured in RPMI 1640 medium supplemented with 10% fetal bovine serum (FBS) plus 100 U/ml penicillin and 100 μg/ml streptomycin. All reagents for culture were obtained from Invitrogen (Carlsbad, CA).

### Cell adhesion assay

Cells were seeded in 6-cm dishes and cultured with RPMI 1640 containing 10% fetal bovine serum (FBS). Matrigel was 1:100 diluted with serum-free RPMI 1640 medium and added into 96-well plate to allow to coat overnight at 4°C. Next day, cells were trypsinized and collected in serum-free medium supplemented with indicated doses of SW. After drug pre-treatment for 15 min (for T24 cells) or 30 min (for MDA-MB-231 and HepG2 cells), cell suspension was then supplemented with 0.5% FBS before being seeded into matrigel pre-coated 96-well plates at the density of 2×10^4^ per well and allowed to adhere for 2 h. After removing non-adherent cells by PBS, adherent cells were photographed with the CloneSelect Imager system (Molecular Devices, Sunnyvale, CA). Nine microscopic fields of each well were randomly captured and cells were counted using Image Pro Plus software.

### Transwell migration/invasion assay

For migration assay, cells were seeded in 10-cm dishes. Next day, cells were harvested in serum-free medium and devided into three equal parts. After each part was supplemented with indicated doses of SW, half of cell suspension was added into the upper well of the transwell insert (Corning Inc, Corning, NY) at the density of 1×10^4^ (for T24 and HepG2 cells) or 5×10^3^ (for MDA-MB-231 cells) per well. The lower well was added with RPMI 1640 containing 10% FBS medium as chemotactic attractors. The remaining half of cell suspension was then seeded into 96-well plates for viability assay. After 12 h’s incubation, cells that have penetrated into bottom side of the transwell membrane were stained with crystal violet and photographed under microscope. 14 microscopic fields were randomly captured and counted. To evaluate the effect of TGF-β1 on cell migration, cells were starved with RPMI 1640 containing 0.5% FBS for 24 h and then collected by trypsinization. Cell suspension were devided into four equal parts and each part was supplemented with solvent, 5 ng/ml TGF-β1, 1.5 μg/μl SW or SW plus TGF-β1. After adding half of drug-containing cell suspension into the upper well of transwell insert, the remaining cell suspension was added into 96-well plates. Both plates were incubated for 12 h. The transwell plates were used for migration assay and the 96-well plates for cell viability assay. For invasion assay, a layer of matrigel (1:4 dilution in serum free medium) was pre-coated on the upper well of transwell insert before adding cells, and cells were incubated for 36 h before crystal violet staining. The rest procedures were the same as migration assay.

### Cell wound healing assay

Two parallel lines were drawn on the back side of the 12-well plates with a marker pen before cell seeding. Next day, the culture medium was removed and replaced by 0.5% FBS/RPMI 1640 medium. 24 h later, a straight wound line that was perpendicular to those parallel lines was drawn across the attached cell layer with pipette tips. Therefore, the wound gap between two parallel lines was marked. After removing the floating cells with PBS, indicated doses of SW were added in each well. Pictures were taken of the marked gap every 6–8 h till the wound closed up. The wound gaps were digitally quantified using Image Pro Plus software.

### Animal model

7- to 8-week-old female Balb/c nude mice (Vital River Laboratories, Beijing, China) were injected through the tail vein with 1×10^6^ MDA-MB-231 cells. Next day, mice (n = 8 for each group) were orally administrated with 72.7 mg SW (prepared in PBS, equals to 1 g of SGR) or PBS per day. After two weeks, the mice were kept for another 3 weeks before being sacrificed for lung and liver collection. Hematoxylin and eosin (H&E) staining was utilized to monitor and count metastatic foci in lung or liver.

### Measurement of cell proliferation

Cells treated with SW or solvent were seeded into 96-well plates and incubated. Cell confluence rate was quantified using CloneSelect Imager system.

### Western blot

Cells exposed to SW for indicated times were collected in lysis buffer containing 50 mM Tris-HCl (pH 7.0), 150 mM NaCl, 2 mM EDTA, 1% SDS, 2 mM Dithiothreitol (DTT) and 1× protease inhibitor cocktail (Roche, Mannhelm, Germany) and then sonicated for 30 s on ice. Protein concentration was determined by BCA kit (Pierce, Rockford, IL). Cell lysates (30 μg per sample) were separated on 8%-15% SDS-PAGE before being electrotransferred to nitrocellulose membranes. After blocking with 5% nonfat milk in 0.1% Tween 20/TBS (TBST), membranes were probed with primary antibodies overnight at 4°C and then reprobed with HRP-labeled secondary antibodies for 45 min at room temperature (RT). Signals were detected by an enhanced chemoluminescence system from Pierce (Rockford, IL).

### Immunofluorescence

Cells were seeded on sterilized coverslips. Next day, SW was added and cells were incubated for indicated time. At the end of the experiment, cells were fixed with 4% paraformaldehyde for 20 min at RT before being permeabilized with 0.1% Triton-X-100 in PBS for 4 min. After blocking with 5% BSA/PBS for 1 h, cells were incubated with anti-vinculin antibody (1:100) overnight at 4°C and then TRITC-conjugated anti-mouse second antibody and FITC-labeled phalloidin (Sigma) mixtures for 1 h at RT. Nuclei were stained with DAPI before capturing pictures through Leica TCS SP5 laser confocal microscope.

### Analysis of focal adhesion

The intensity of vinculin signal and the area of focal adhesion were analyzed by Adobe Photoshop CS5 and Image Pro Plus software. Firstly, Vinculin-stained images were de-saturated, inverted and de-noised using Photoshop software to produce adhesion footprints. Next, these vinculin-containing footprints were further selected out by thresholding in Image Pro Plus software. Through calibrating, the area and intensity of vinculin-containing footprints was quantified using the “count/size” command. A total of 50 cells were analyzed in each group.

### Microarray analysis and real-time RT-PCR

RNA was extracted from HepG2 cells treated with SW (1.5 μg/μl) using Trizol reagent (Invitrogen) and cDNA was synthesized using reverse transcriptase system (Promega, Madison, WI). Gene expression profiles were examined by ShangHai Biotechnology Corporation (Shanghai, China) using Affymetrix Human Gene 1.0st microarrays. Microarray data has been deposited in NCBI Gene Expression Omnibus (GEO) (accession no. GSE58201). After Robust Multi-array Average (RMA) normalization, *P* value < 0.05 and the Fold-Change threshold > 1.5 were identified to be statistically significant alterations. KEGG, GO and Gene Cards (http://www.genecards.org/) were utilized to select out genes that was related to cell adhesion, migration and invasion. Next, these genes were put into STRING database for signaling pathway searching. Real-time RT-PCR was used to validate the results of microarray and carried out according to the manufacturer’s instruction (SYBR, TOYOBO). Expression levels of all genes were normalized through *GAPDH* gene and the 2^−ΔΔCT^ method was used to calculate relative gene expression. The primers for real-time RT-PCR (*TGFBR1*, forward, 5'-CCTGGGATTTATAGCAGCAGAC-3’, reverse, 5’-TGACACCAACCAGAGCTGAG-3’; *NTS*, forward, 5’-ATTAGTAAAGCACATGTTCC-3’, reverse, 5’-CTCCCAGTGTTGAAAAGCCC-3’; *ID1*, forward, 5’-GAGCTGAACTCGGAATCCGAAG-3’, reverse, 5’-GATCGTCCGCAGGAACGCATGC-3’; *ID2*, forward, 5’-TCAGCCTGCATCACCAGAGA-3’, reverse, 5’-CTGCAAGGACAGGATGCTGATA-3’; *ID3*, forward, 5’-CTGAGCTTGCTGGACGACA-3’, reverse, 5’-ATGTAGTCGATGACGCGCTGTA-3’) were synthesized by GenePharma (Shanghai, China).

### Statistical analysis

Data analysis was performed using SPSS 13.0 (SPSS, Inc., Chicago, IL). Two-tailed student’s t test and ANOVA were used to determine the significance of differences between different experimental groups.

## Results

### SW boosts cell adhesion

**Fig 1:**
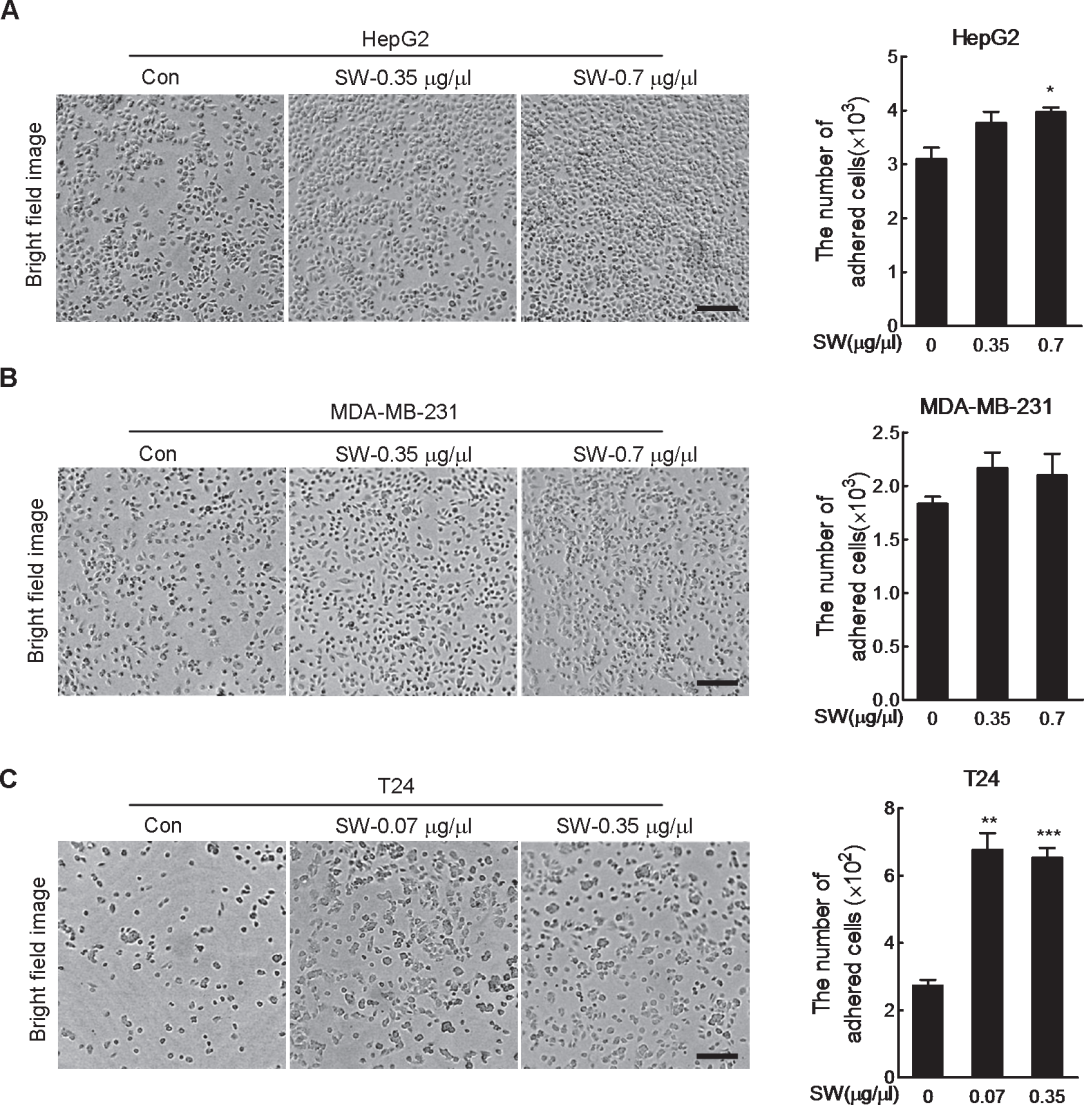
SW promotes cell adhesion. Left panels: HepG2 (A), MDA-MB-231 (B) and T24 cells (C) were pretreated with indicated doses of SW for 15 or 30 min before seeding into the matrigel-coated wells, and 2 h later, adhered cells were counted after removing the floating cells by PBS. Representative images were displayed. Scale bar, 200 μm. Right panels: quantification of the data in the left panel, shown were composite results of three independently experiments with triplicate. Columns, mean; bars, SD.

To test the effect of SW on cancer cells’ capacity to bind to extracellular matrix, we performed cell adhesion assay using hepatoma cell line HepG2 (Fig. 1A), breast cancer cell line MDA-MB-231 (Fig. 1B) and bladder cancer cell line T24 (Fig. 1C). It was found that the adhesion of HepG2 cells to matrigel was increased by 0.7 μg/μl of SW (Fig. 1A), while effect of same concentration of SW on the adhesion of MDA-MB-231 cells was marginal (Fig. 1B). To the contrary, the increase in adhesion was more evident in T24 cells and the highest adhesion was achieved by as low as 0.07 μg/μl of SW (Fig. 1C).

### SW inhibits cancer cell migration

**Fig 2:**
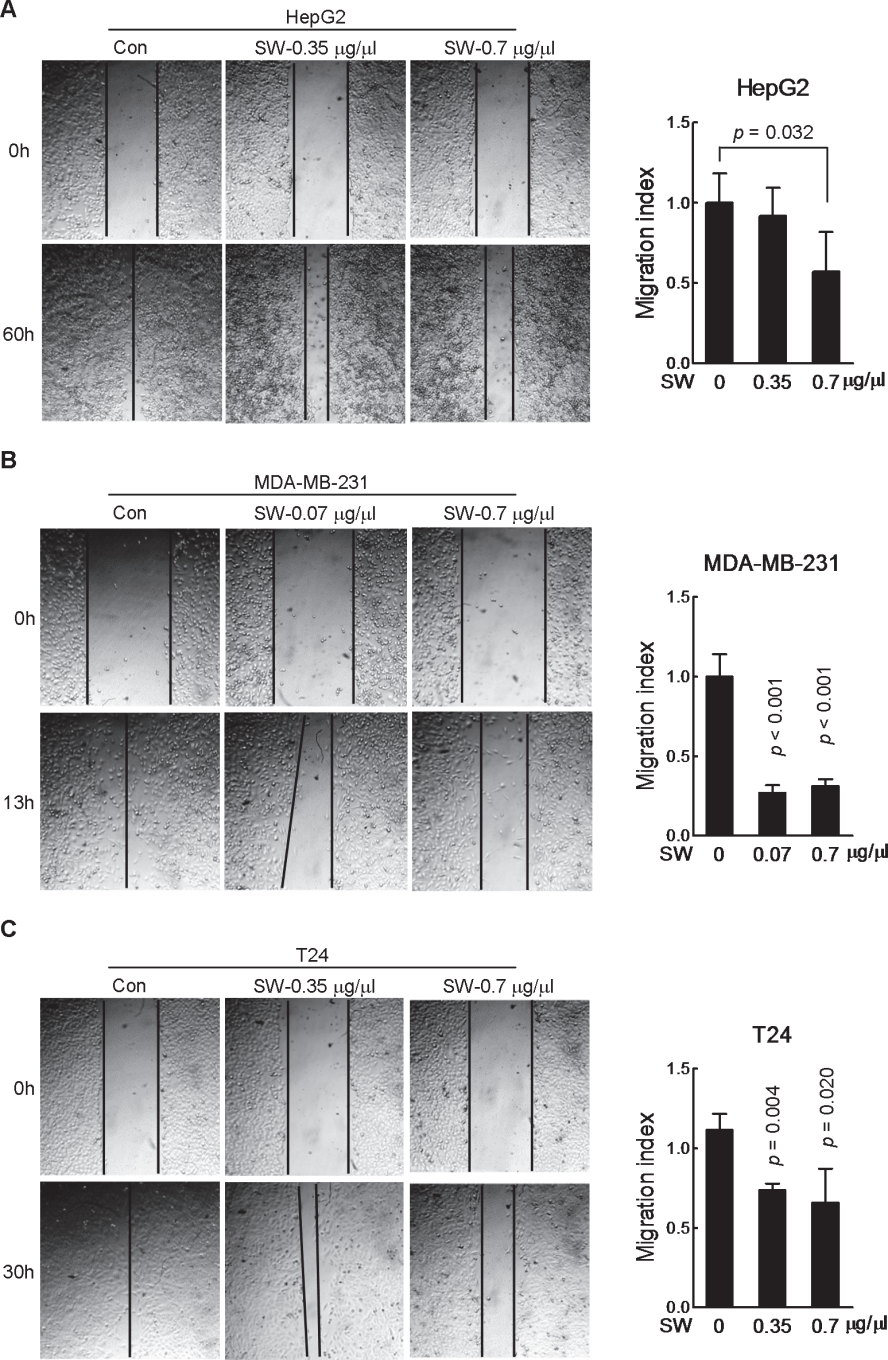
SW inhibits cell scratch wound healing. *In vitro* scratch assay was used to evaluate the effect of SW on the migration of HepG2 (A), MDA-MB-231 (B) and T24 cells (C). Representative images were displayed in left panel; quantification of the data in left panel was shown in right panel, shown were composite results of three independently experiments with triplicate parallel samples. The migration index represents migration speed in relative to control group. Columns, mean; bars, SD.

**Fig 3:**
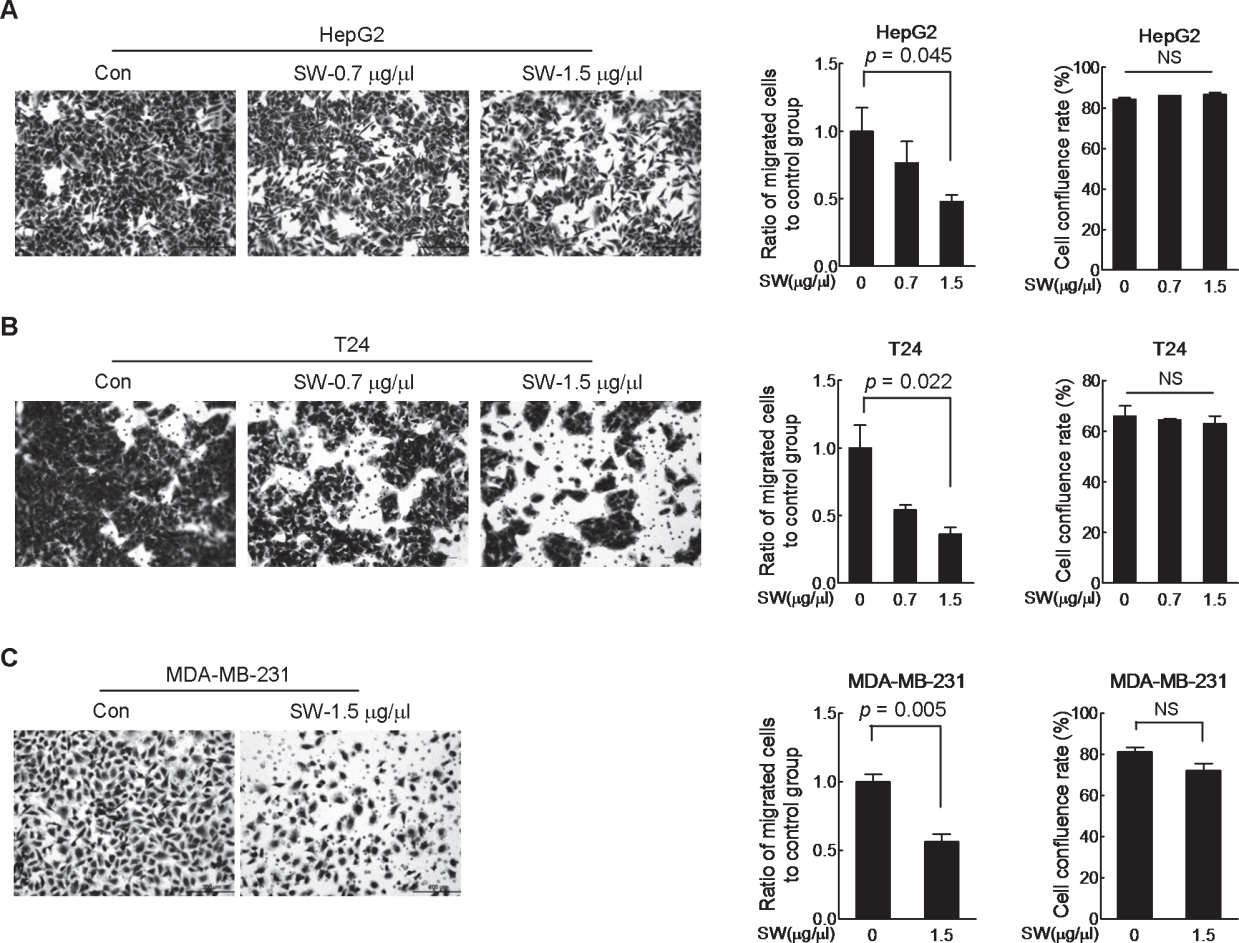
SW inhibits cell migration. HepG2 (A), T24 (B) and MDA-MB-231 (C) cells were seeded into the transwell insert in the presence of indicated doses of SW for 12 h. Cells reaching the bottom side of the transwell membrane were stained and representative images were displayed in the upper panel. Scale bar, 200 μm. The data in left panel were quantified and shown in the middle panel, and the cell viability was reflected by cell confluence rate in the right panel. Shown were composite results of two independently experiments. Columns, mean; bars, SD; NS, not statistically significant.

Next, we performed *in vitro* scratch assay. Cells were pre-incubated in 0.5% FBS medium for 24 h prior to the introduction of scratch in order to exclude the impact of the FBS on cell proliferation. The wound healing was monitored at regular intervals till it closed up. We observed that SW led to a slow-down of migration in HepG2, MDA-MB-231 and T24 cells (Fig. 2A, B and C), with MDA-MB-231 cells showing the most obvious inhibitory effect (Fig. 2B). We noticed that it required different time for different cell lines to heal the wounds. Additionally, same concentration of SW exhibited distinct inhibition on wound healing in different cell lines. This could be explained by cell linage specificity at migration. Considering the roughness and subjectivity of the scratch assay, we also used transwell migration assay. The cells that succeeded in squeezing through the micro-holes of the base membrane in the SW-treated group were markedly less than those of control group (Fig. 3A, B and C, left and middle panels), with little influence on cell viability (Fig. 3A, B and C, right panels). These results pointed to the inhibitory action of SW on cancer cell migration.

### SW inhibits cancer cell invasion *in vitro* and metastasis *in vivo*

**Fig 4:**
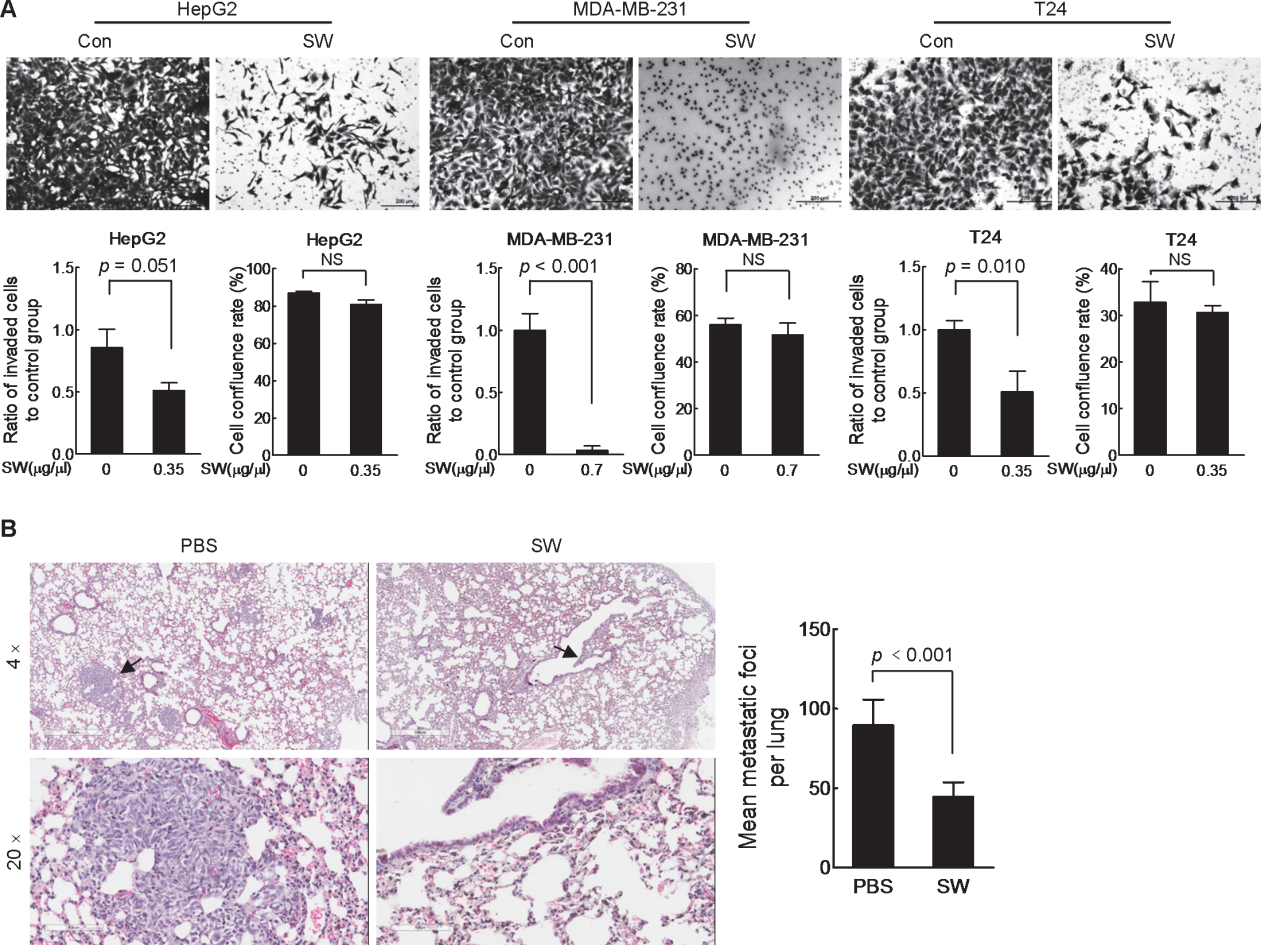
SW inhibits cell invasion *in vitro* and metastasis *in vivo*. (A) The transwell chambers were pre-coated with 60 μl matrigel dilution (1:4 in serum-free medium) before seeding cells. Cells were incubated with indicated doses of SW for 36 h before staining. Representative images were displayed in the upper panel. Scale bar, 200 μm. The data of migration and cell viability were quantified and shown respectively in lower panel, shown were composite results of two independently experiments with triplicate. Columns, mean; bars, SD; NS, not statistically significant. (B) Left panel: representative pictures of H&E stained lung tissues in PBS- and SW-treated group. Paraffin-embedded lungs were sectioned at 30-μm intervals and > 10 MDA-MB-231 cancer cells were identified as metastatic foci. Magnification, 4× (upper) and 20× (lower). Right panel: quantification of lung metastatic foci in PBS- and SW-treated mice group (n = 8 for each group). Columns, mean; bars, SD.

We also tested the effect of SW on cancer cell invasion. Still, we used the same transwell device plus loading a layer of matrigel over the base membrane. As expected, SW treatment prevented all three cell lines from invading across the matrigel to reach the bottom side of the base membrane (Fig. 4A, upper panel and lower left panel). Additionally, 36 h exposure to indicated doses of SW exerted insignificant inhibition on cell proliferation (Fig. 4A, lower right panel). To further evaluate the effect of SW on metastasis *in vivo*, MDA-MB-231 metastatic mice model was used. As shown in Fig. 4B, oral administration of SW for 2 weeks markedly decreased the number of lung metastatic foci. No liver metastatic foci were observed in both groups (S1 Fig.).

### SW boosts focal adhesion

**Fig 5:**
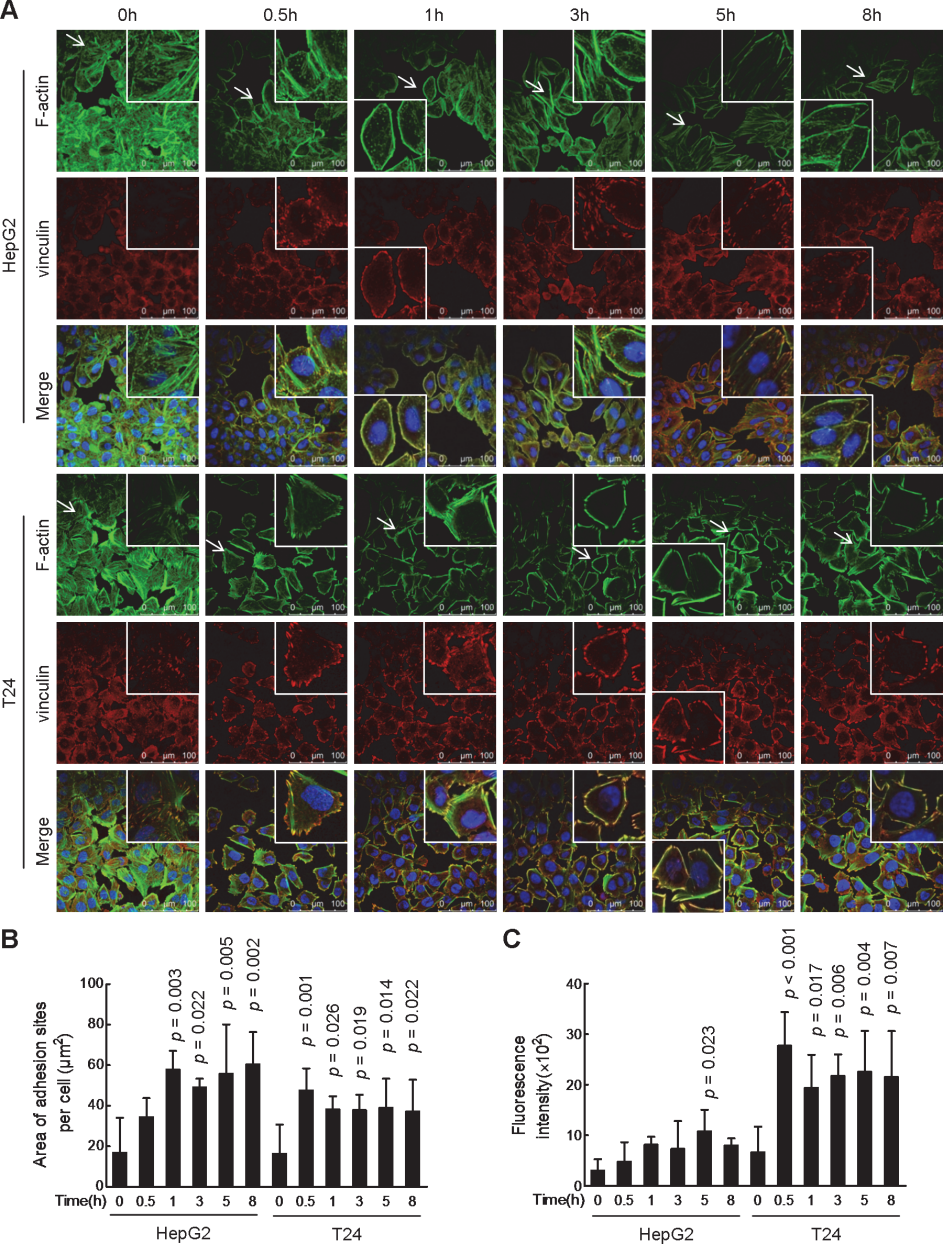
SW increases size and fluorescence intensity of focal adhesion. (A) HepG2 and T24 cells were treated with SW (3.5 μg/μl) for indicated times and then immuno-stained with F-actin (FITC-phalloidin) and vinculin (red). Nuclei were counterstained by DAPI (blue). Representative images were displayed. Scale bar, 100 μm. (B) The area of adhesion sites per cell (where F-actin and vinculin merged). Shown were composite results of two independently experiments (n = 50 cells). Columns, mean; bars, SD. (C) The fluorescence intensity of vinculin in the adhesion patches per cell. Shown were composite results of two independently experiments (n = 50 cells). Columns, mean; bars, SD.

It was reported that the number and size of focal adhesions were inversely proportional to migration speed [24]. For this we performed dual fluorescent staining of F-actin and vinculin to assess the effect, if any, of SGR on focal adhesion [20,24,25]. After exposure to SW for 0.5 h, HepG2 and T24 cells began to exhibit stronger vinculin signal mainly around the cell periphery, displaying a fascicle-like or spot-like shape (Fig. 5A). Correspondingly, large stress fibers across the cell body were lost, with F-actin fibers gradually shifting to cell periphery and co-localizing there with vinculin as well. We quantified the areas and fluorescent intensities of adhesion sites. As shown in Fig. 5B, the adhesion area per cell elevated at 0.5 h and reached plateau at 1 h in SW-treated HepG2 cells, while it already achieved peak at 0.5 h after SW treatment in T24 cells. Similar results were obtained in intensity quantification (Fig. 5C).

### SW antagonize TGF-β1-induced up-regulation of genes related to invasiveness

**Fig 6:**
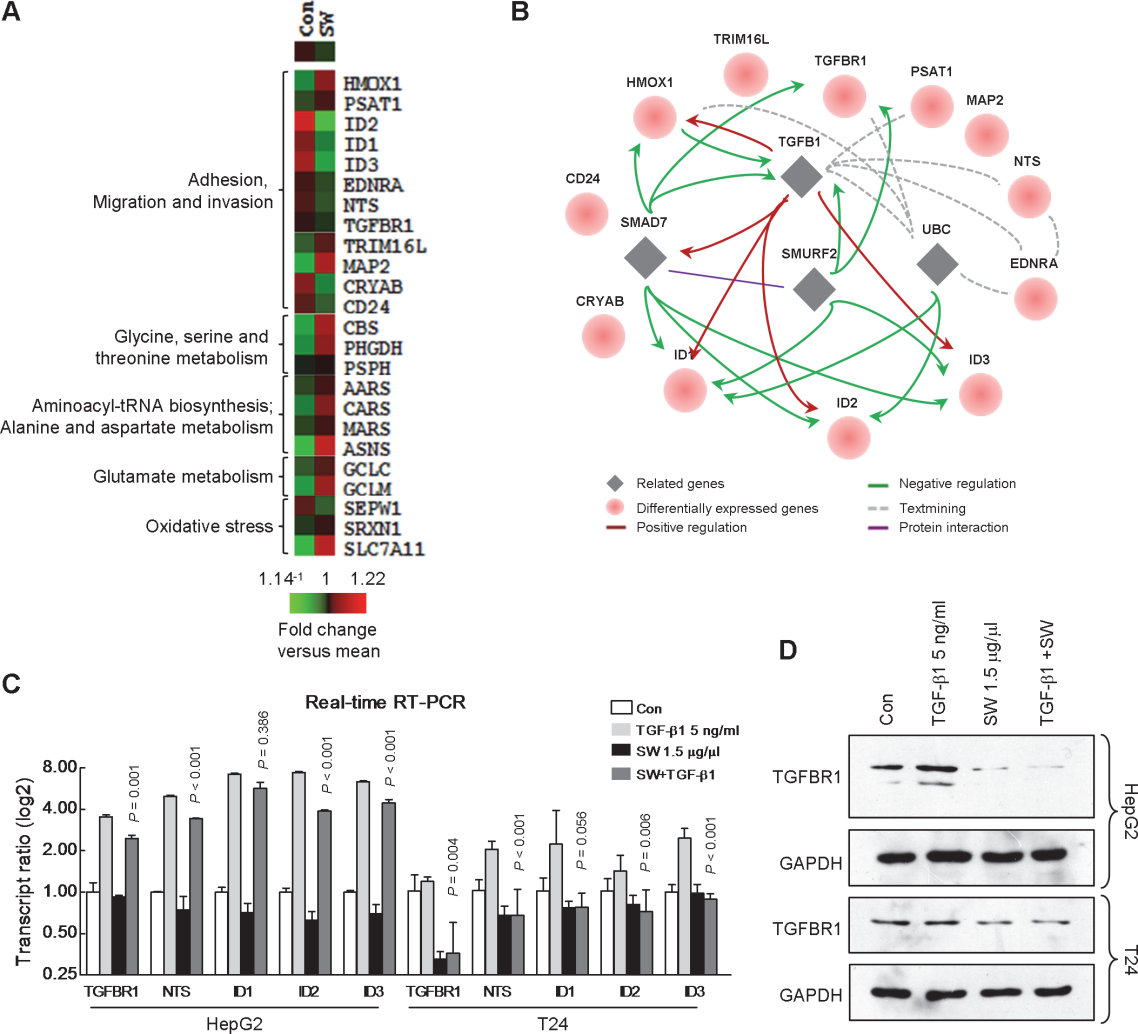
Microarray analysis result indicated repression of TGF-β1 pathway in SW-treated cells. (A) The heat map of functional enrichment of differentialy expressed genes from the HepG2 chip result. Cluster 3.0 and TreeView software were used for generating the heat map. Red: up-regulation versus mean; green: down-regulation versus mean. (B) The regulatory network among the genes related to adhesion, migration and invasion in (A) using the STRING database. (C) Real-time RT-PCR verification of the genes in HepG2 and T24 cells treated with TGF-β1 with or without SW. Shown were composite results of three independently experiments with triplicate. Columns, mean; bars, SD. (D) Effect of SW on TGF-β1-induced up-regulation of TGFBR1 was evaluated by immunoblotting. Shown were representative results from 3 independent experiments.

**Fig 7:**
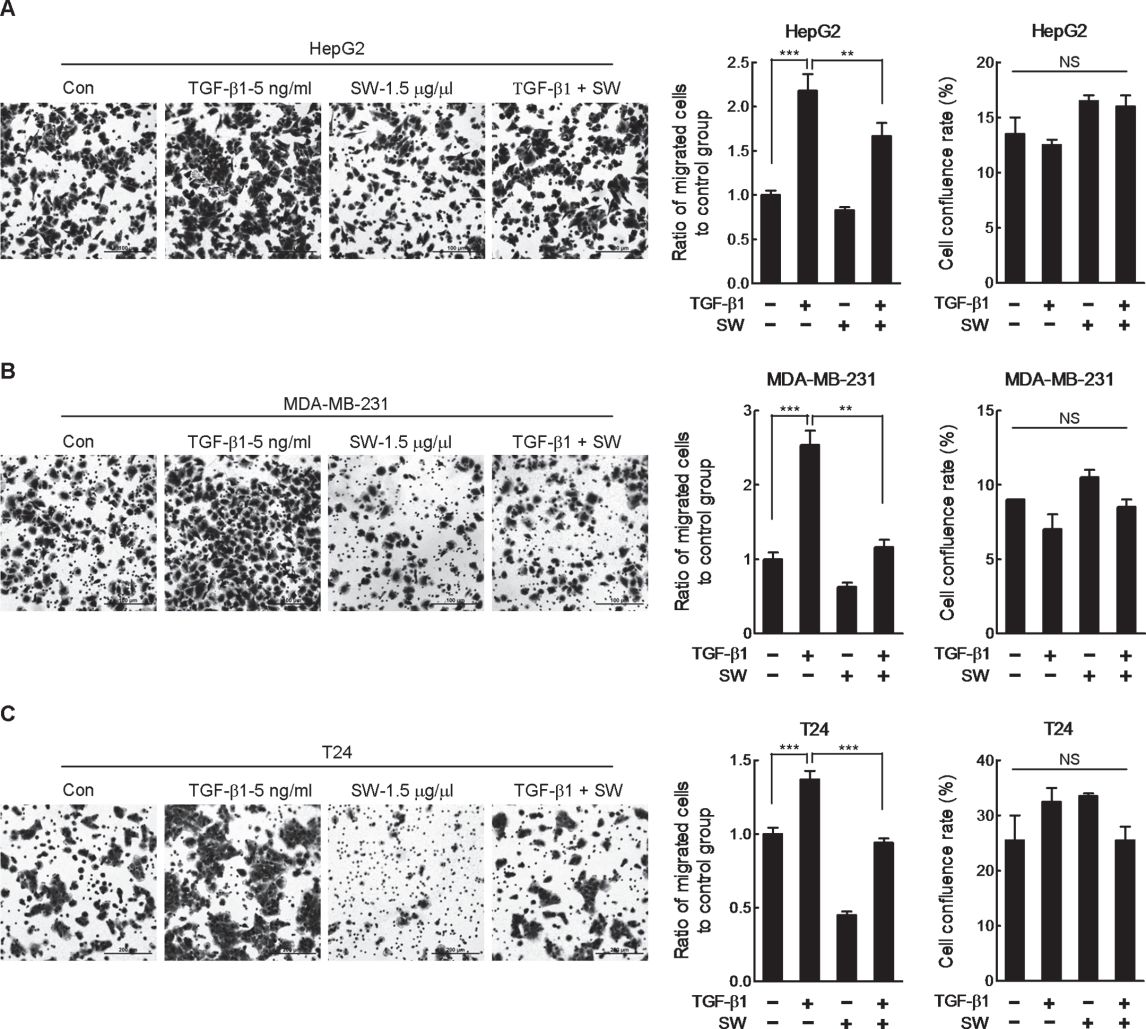
SW abrogates TGF-β1-induced boost in migration. HepG2 (A), MDA-MB-231 (B) and T24 (C) cells were seeded into the transwell inserts in the presence of TGF-β1 plus SW. 12 h later, Cells were stained and pictured. Representative images were displayed in the left panel. Scale bar, 100 μm. The data of migration were quantified and shown in the middle panel. The cell viability was reflected by cell confluence rate in the right panel. Shown was composite results of two independently experiments with triplicate. Columns, mean; bars, SD; NS, not statistically significant.

To get a deeper understanding of the possible mechanism underlying all these results, we performed a gene expression microarray analysis with SW-treated HepG2 cells. We found there were 118 genes with more than 1.5 fold expression change in the cells treated with SW, some of them were related to cell motility, metabolism, and oxidative stress (Fig. 6A). Next, we analyzed a subset of genes associated with cell invasiveness using STRING database. As shown in Fig. 6B, most of these 12 genes were directly or indirectly regulated by the TGF-β1 pathway, indicating that TGF-β1 pathway might be involved in SW-regulated phenotypes. Real-time RT-PCR was performed to evaluated expression levels of 5 genes, i.e., *TGFBR1*, *NTS*, *ID1*, *ID2*, and *ID3* in HepG2 and T24 cells treated with TGF-β1 with or without SW. SW alone decreased levels of *NTS*, *ID1*, *ID2*, and *ID3* in HepG2 cells and *TGFBR1*, *NTS*, *ID1*, and *ID2* in T24 cells. On the other hand, TGF-β1 significantly elevated mRNA levels of these genes in both cell lines (Fig. 6C), confirming that these genes are downstream of TGF-β1 signaling. However, majority of TGF-β1-induced increase in gene expression was counteracted by concomitant treatment with SW, except for *ID1* in HepG2 cells (Fig. 6C). Additionally, we observed up-regulated protein levels of TGFBR1 in TGF-β1-treated HepG2 and T24 cells, which was reversed by SW (Fig. 6D). To further confirm the inhibitory effect of SW on TGF-β1 signaling, we evaluated the effect of SW on TGF-β1-induced migration. As shown in Fig. 7, TGF-β1-promoted migration was markedly abrogated by SW in HepG2, MDA-MB-231 and T24 cells, while cell viability was unaffected by SW (Fig. 7A, B and C).

## Discussion

SGR, also known as Sarsaparilla, was reported to show anticancer potential [8,21]. Several SGR-containing herbal formulae used in Southeast Asia were proven to inhibit cancer *in vitro* and *in vivo* [20,26]. The majority of previous studies focused on the inhibitory effect of SGR on cancer cell growth, and only a few evaluated its effect on the invasiveness of cancer cells. A SGR-containing herbal formula was shown to suppress lung carcinoma metastasis [27]. Compounds isolated from SGR, i.e. 5-O-caffeoylshikimic acid, taxifolin and astilbin, were shown to have inhibitory effect on adhesion or/and migration of immune cells [22]. In previous study, we found the cell growth inhibition of SGR was a relatively long-term effect (> 24 h) under high doses [21]. In the present study, we, for the first time, reported the inhibitory effect of SGR extract on the invasiveness of three cancer cells, likely through the suppression of TGF-β1 pathway. It should be mentioned that all the *in vitro* experiments were performed with low doses of SW within short term to minimize the bias resulted from SW-induced growth inhibition.

Focal adhesion is the basic structure module unit of cell adhesion, any changes in its structure or composition might influence the adhesion strength of cells on the whole [24,25]. The formation of focal adhesion goes through several stages, including nascent adhesion, focal complex and then focal adhesion [25]. The size and maturity of focal adhesions were reported to be inversely correlated with cell migration speed [24]. In the present study, we observed a gradual shift of the adhesion patches from the cell body to cell perimeter in SW-treated HepG2 and T24 cells, with stronger vinculin signal and larger adhesion size on the cell cortex. This phenomenon was, at least in part, due to a space- and site-directed shift of adhesion-related proteins, especially in T24 cells. Interestingly, SW-induced large and firm peripheral adhesions resembled the focal adhesion kinase (FAK)-deficient cells [25,28]. Additionally, cells lacking either tyrosine phosphatases like Shp-2 or the Src kinases also exhibited a similar adhesion pattern [24,29]. Therefore, an impairment of FAK-Src pathway might mediate this SW-induced effect, which requires further investigation.

The chip result indicated a repression of TGF-β1 signaling in SW-treated cells. Genes including *HMOX1* [30,31], *PSAT1* [32], *TGFBR1* [33], *EDNRA* [34,35], *NTS* [36,37], *ID1* [38,39], *ID2* [40,41] and *ID3* [42,43], all related to cell adhesion, migration and invasion, were either directly or indirectly regulated by TGF-β1 signaling pathway. The inactivation of TGFBR1’s kinase domain could prevent TGF-β1 signaling from propagating down [33]. Meanwhile, abrogation of TGF-β1 signaling using dominant negative TGFBR could block epithelial-mesenchymal transition (EMT) switch *in vivo* [44], suggesting that suppression of TGFBR might provide a feasible means to repress TGF-β1 signaling. In the present study, TGF-β1-induced TGFBR1 was diminished by SW. Additionally, SW abrogated TGF-β1-induced boost in migration, confirming the inhibitory effect of SW on TGF-β1 pathway.

It should be mentioned that TGF-β1 signaling was found to increase cell adhesion and promote focal adhesion [45], therefore SW-induced cell adhesion and focal adhesion may rely on distinct mechanisms. This raises the possibility that SW-inhibited cancer cell invasiveness could be the integrated effects of several signaling events. Other pathways were also predicted to be affected by SW in the microarray analysis, such as metabolism and oxidative stress. Recent studies highlight the contributions of deregulated metabolism and oxidative stress to cancer cell invasiveness [46,47]. Whether SW could utilize these pathways to regulate cancer cell invasiveness deserve further investigation.

Together, we found SGR could promote cell adhesion possibly by increasing the size and strength of focal adhesions, and inhibit migration and invasion of HepG2, MDA-MB-231 and T24 cells. Repression of TGF-β1 signaling partially contributes to SW-induced migration inhibition. Although these results were obtained from a subset of cancer cell lines, the novel anticancer function of SGR may provide a possible molecular basis for its future clinical application in cancer treatment.

## Supporting Information

S1 Fig No metastatic foci was observed in liver of mice. Representative pictures of H&E stained liver tissues in PBS- and SW-treated group. Paraffin-embedded livers were sectioned at 30-mm intervals and ˃10 MDA-MB-231 cancer cells were identified as metastatic foci. Scale bar, 200 mm, magnification, ×10.
(TIF)
