# Supplementary figures and images for: Sarsaparilla (Smilax Glabra Rhizome) Extract Inhibits Migration and Invasion of Cancer Cells by Suppressing TGF-β1 Pathway
Source: PLoS One. 2015 Mar 5;10(3):e0118287. doi: 10.1371/journal.pone.0118287 (PMC4351248; doi:10.1371/journal.pone.0118287)

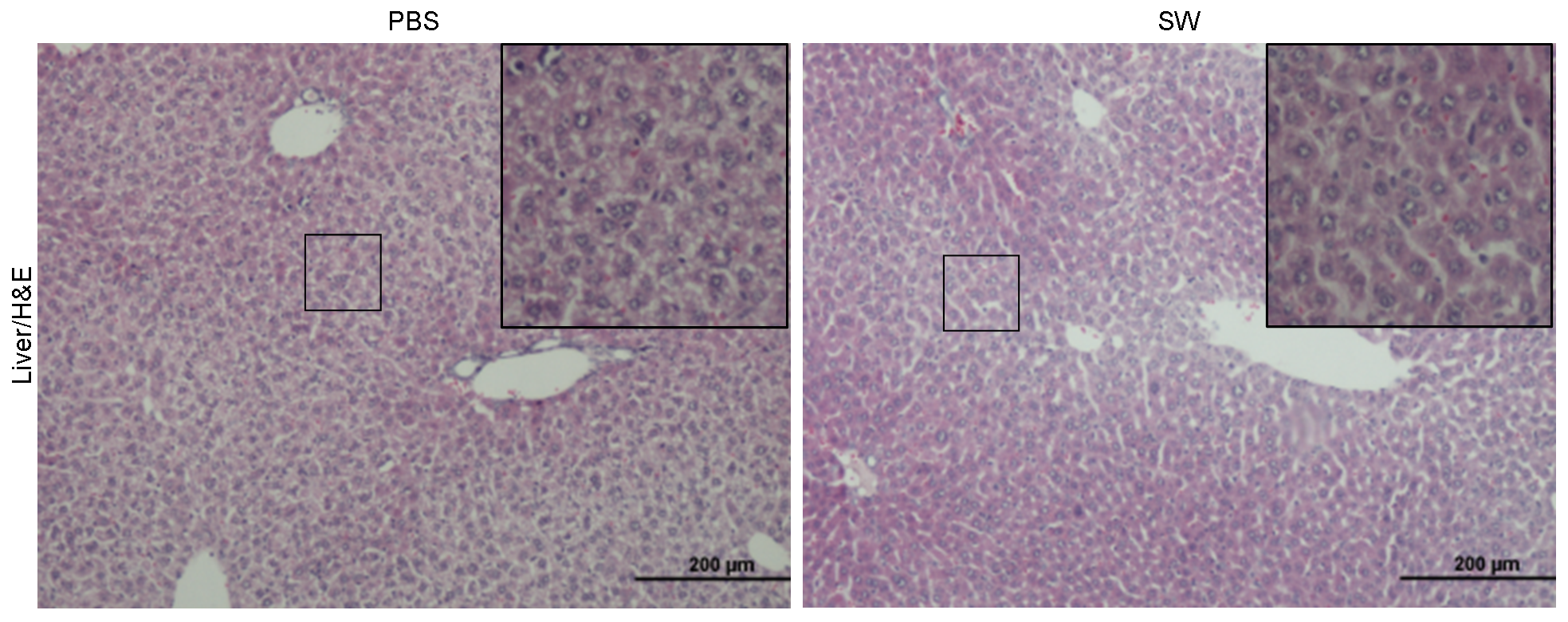

Supplement: S1 Fig — Representative pictures of H&E stained liver tissues in PBS- and SW-treated group. Paraffin-embedded livers were sectioned at 30-mm intervals and ˃10 MDA-MB-231 cancer cells were identified as metastatic foci. Scale bar, 200 mm, magnification, ×10. (TIF) [file pone.0118287.s001.tif]
